# Accuracy of Bowel Ultrasound for Detecting Inflammatory Bowel Disease at a Tertiary Hospital in Central South Africa

**DOI:** 10.1002/jcu.70041

**Published:** 2025-08-15

**Authors:** Amanda Hendricks, Henra Muller

**Affiliations:** ^1^ Department of Clinical Sciences, Faculty of Health and Environmental Sciences Central University of Technology Bloemfontein Free State South Africa

**Keywords:** bowel biopsy, bowel ultrasound, bowel ultrasound parameters, colonoscopy, Crohn's disease, inflammatory bowel disease, ulcerative colitis

## Abstract

**Background:**

To prevent complications, early diagnosis and treatment of inflammatory bowel disease (IBD) are crucial. The gold standard for diagnosis is colonoscopy with biopsy, which is invasive and costly. Bowel ultrasound (BUS) is a non‐invasive and accurate diagnostic imaging tool. Thus, the aim of this study was to evaluate the accuracy of BUS in detecting IBD, compared to the gold standard of colonoscopy with biopsy.

**Objectives:**

A BUS imaging protocol for IBD was used to make a preliminary diagnosis of IBD with a BUS scan. The preliminary diagnoses were compared to the confirmatory diagnoses that used histology.

**Method:**

A radiologist made a preliminary diagnosis using the instrument BUS parameter measurements. Fisher's exact test (*p* = 0.05) was performed to determine if an association existed between the preliminary IBD diagnosis and confirmation. The accuracy of BUS diagnosis was calculated for the individual parameters and the preliminary ultrasound diagnosis.

**Results:**

Of the participants, 47 (61%) were diagnosed with IBD, and 30 (39%) were negative. The majority had thickened bowel walls and increased bowel wall vascularity. The BUS parameters of bowel wall thickness and vascularity accuracy were above 80%. Bowel wall stratification, haustration, fatty wrapping, peristalsis, mesenteric lymph nodes, and free fluid ranged between 66% and 71%. The overall accuracy of BUS parameters exceeded 80%.

**Conclusion:**

The potential of using BUS parameters as a sifting or screening tool should be considered in the workup to expedite the diagnosis of IBD.

**Contribution:**

The identification and description of BUS parameters for ultrasound diagnosis of IBD may facilitate the diagnosis and monitoring of IBD.

## Graphical abstract

Bowel wall thickness, as measured by bowel ultrasound, demonstrates high diagnostic accuracy in evaluating patients with suspected inflammatory bowel disease (IBD). When combined with other bowel ultrasound parameters, this non‐invasive tool can serve as an effective screening method to accelerate IBD diagnosis.

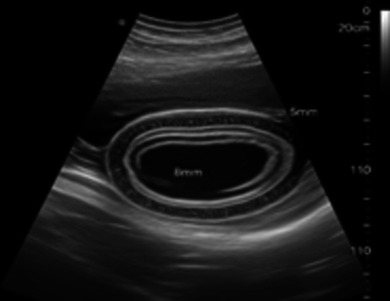

## 1 Introduction

Inflammatory bowel disease (IBD) is a chronic gastrointestinal condition characterized by exacerbations that can, over time, lead to complications such as stenosis and perforation (Wilson 2020; Furfaro et al. 2021; Windsor et al. 2023). The exact cause of IBD remains unknown, but it is believed that it may result from a combination of immune system dysfunction, genetics, autoimmune conditions, and environmental factors (Furfaro et al. 2021; Tavakoli et al. 2021; Saoji et al. 2024). The incidence and prevalence of IBD have increased significantly over the last decades and affect over 6 million people globally (Watermeyer et al. 2020; Silangcruz et al. 2021). IBD may occur at any stage of life and is more common in the White population and people with a family history of IBD (Maconi et al. 2018). Literature shows that the prevalence of IBD in the White population with a family history of IBD is higher (26%–33%) than in African American (9%–18%), Hispanic (9%–16%) or Asian (5.9%) populations (Santos et al. 2018; Hodges and Kelly 2020). IBD is a well‐recognized disease in the White population in South Africa, though it is relatively rare in Indigenous African populations (Chonco et al. 2021).

The most common types of IBD are Crohn's disease and ulcerative colitis. Crohn's disease causes inflammation in the lining of the digestive tract and can develop in any part of the digestive system. Ulcerative colitis, in turn, causes chronic inflammation, mainly in the colon and the rectum (Zhang and Li 2014). Patients with IBD present with clinical symptoms such as abdominal pain, bloody stools, fatigue, diarrhea, weight loss, iron deficiency, and other features of malabsorption, which may affect a patient's quality of life (Silangcruz et al. 2021; Muradali and Goldberg 2015; Perler et al. 2019).

Early diagnosis and treatment of IBD are essential to prevent complications that can significantly affect patient morbidity and mortality. To diagnose and confirm IBD, patients who exhibit clinical symptoms undergo clinical evaluations, biochemical assessments, endoscopic procedures (such as colonoscopy with biopsy) and various imaging techniques. Currently, colonoscopy with biopsy is considered the gold standard for diagnosing IBD (Fodor et al. 2021). While colonoscopy with biopsy provides crucial histological confirmation, other imaging modalities can complement the diagnostic process. Colonoscopy is not without its drawbacks: it is costly, invasive, and carries risks, including bowel perforation (Hajiani et al. 2018). Additionally, it may not effectively assess the entire bowel or evaluate complications associated with IBD, and many patients find the procedure challenging to tolerate (Maaser et al. 2019).

Some of the many challenges relating to IBD that the healthcare systems in resource‐limited settings might face include the need for access to diagnostic facilities (endoscopy and histopathology facilities), underreporting or missed diagnosis of IBD cases, financial challenges related to medical treatment for patients with IBD, and the difficulty of differentiating between the diagnoses of Crohn's disease and intestinal tuberculosis (Hodges and Kelly 2020; Watermeyer et al. 2023). BUS is a diagnostic imaging modality that yields similar information as colonoscopy and magnetic resonance enterography (MRE) by considering different parameters. BUS is a non‐invasive, radiation‐free, accurate, cost‐effective, fast, reproducible, and widely available imaging tool with high sensitivity (up to 85%) and specificity (95%) to diagnose and assess IBD (Kucharzik et al. 2017; Allocca et al. 2018). BUS may serve to expedite diagnosis, decrease the unnecessary use of invasive techniques, and reduce medical costs. BUS offers direct visualization of the colon segments and ileum, which makes assessing the bowel wall layers easier and allows for immediate interpretation and treatment decision‐making (Parente et al. 2002). Compared to other imaging modalities, BUS has the same level of accuracy as colonoscopy and MRE for assessing and monitoring IBD (Allocca et al. 2020). BUS has the same level of accuracy in detecting IBD complications as MRE and computed tomography enterography (CTE) (Hate and Imamura 2022). BUS has the potential to accurately diagnose acute diverticulitis, with high comparative sensitivity (84%–94%) and specificity (80%–93%) compared to CTE (Frias‐Gomesa et al. 2021). Compared to other non‐invasive diagnostic tools, such as fecal calprotectin, BUS offers additional information on the disease extension, location, severity, and complications (Frias‐Gomesa et al. 2021).

To enhance the reliability of BUS in detecting IBD, the European Federation of Societies for Ultrasound in Medicine and Biology (EFSUMB) established standardized ultrasound guidelines (Maaser et al. 2019; Jauregui‐Amezaga and Rimola 2021). These guidelines outline specific techniques and scoring systems for evaluating IBD patients. They recommend using BUS for initial evaluation and for assessing disease extent, activity, and potential complications (Maconi et al. 2018; Maaser et al. 2022). Additionally, to detect relapses or to monitor treatment responses in suspected IBD cases, BUS has been proposed as a potential alternative to colonoscopy. However, the comparative accuracy of BUS versus colonoscopy has not been definitively established (Maconi et al. 2018; Jauregui‐Amezaga and Rimola 2021). Therefore, the aim of this study was to evaluate the accuracy of BUS compared to the gold standard of colonoscopy with biopsy for detecting IBD.

## 2 Research Methods and Design

**FIGURE 1:**
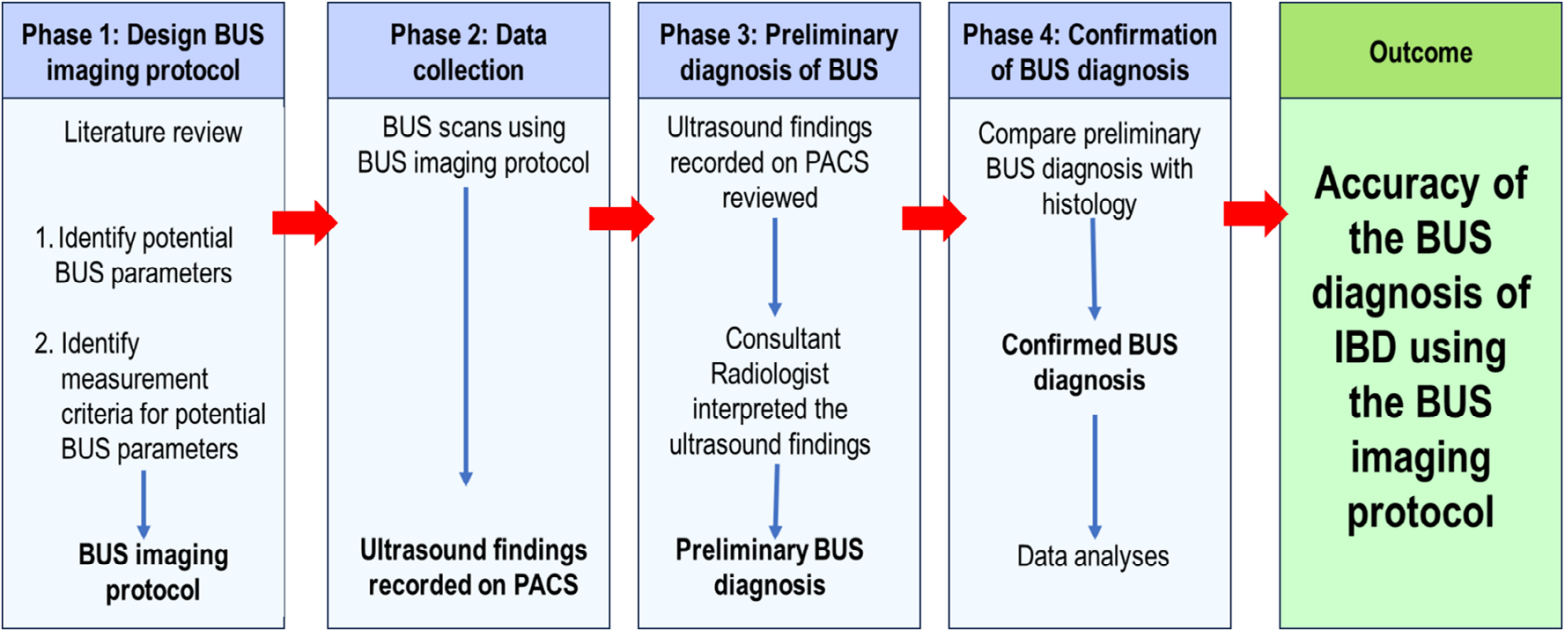
Conceptual framework of the research process of the study.

To ascertain the accuracy of BUS in diagnosing suspected IBD, a prospective quantitative study was conducted on patients from December 2022 to August 2023 at an academic hospital and one private hospital in Bloemfontein, South Africa. The study was conducted over four phases (Figure 1). In the first phase, a BUS imaging protocol was designed to guide the data‐capturing process. In the second phase, the imaging protocol was used by a single experienced certified sonographer to record participant data relating to a preliminary diagnosis of IBD using BUS. In Phase 3, a radiologist reviewed the participant data to make a preliminary IBD diagnosis. Finally, the data comparing the preliminary diagnoses with confirmatory diagnoses using histology (through colonoscopy and biopsy) were used to ascertain the accuracy of BUS for detecting IBD.

### 2.1 Phase 1: Design BUS Imaging Protocol for IBD

In the first phase, a BUS imaging protocol was designed to guide the data‐capturing process for the preliminary diagnosis of IBD with a BUS scan. Peer‐reviewed literature was used to identify potential BUS parameters that could be used to diagnose IBD. In total, eight BUS parameters (bowel wall thickness (BWT), vascularity (identified using color Doppler signals), bowel wall stratification, haustration of the colon, fatty wrapping, peristalsis, mesenteric lymph nodes, and free fluid) were identified from the literature and deemed appropriate for the BUS diagnoses of IBD. Measurement criteria for each of these parameters were also established from the literature.

### 2.2 Phase 2: Data Collection

In the second phase, a transabdominal ultrasound scan, including the bowel, was performed by a single experienced certified sonographer, using the BUS imaging protocol. Prior to the scans, participants were asked to: (1) refrain from eating for a minimum of 6–8 h before the scan to minimize bowel gas and enhance the visibility of the bowel loops, (2) adhere to a low‐gas diet for 1–2 days beforehand by avoiding gassy beverages and foods such as onions and cabbage, as well as refraining from smoking or chewing gum, as these habits can increase intraluminal gas. Shortly before the examination, some participants were given water to drink to aid bowel movement and enhance visualization of the small intestine. The sigmoid colon, descending colon, transverse colon, ascending colon, terminal ileum, proximal small bowel, and rectum were scanned to record participant data relating to a preliminary diagnosis of IBD. The ultrasound findings were recorded and saved on PACS (Picture Archiving and Communication System).

### 2.3 Phase 3: Preliminary Diagnosis of IBD Using BUS Imaging Protocol

In Phase 3, the ultrasound findings, which were recorded and saved on PACS, were interpreted and confirmed by a consultant radiologist. After reviewing the findings, the radiologist generated an ultrasound report of the preliminary BUS diagnosis for each of the participants.

### 2.4 Phase 4: Confirmation of BUS Diagnosis

The IBD diagnoses from preliminary ultrasound reports were compared to histology and colonoscopy findings in the fourth phase. For the colonoscopy procedure, all participants underwent extensive bowel cleansing to ensure optimal mucosal visualization. This preparation included a low‐fiber diet for 2–3 days before the procedure, followed by a 24‐h clear liquid diet. The data from the preliminary ultrasound and the histological and colonoscopy reports were captured electronically in Microsoft Excel. Statistical analyses were performed using SAS Version 9.2. Descriptive statistics, including nominal frequencies and percentages, were calculated for the categorical data, and means, medians, and percentiles were calculated for the numerical data. The Shapiro–Wilk test was used to investigate whether numerical data followed a normal distribution. To compare the accuracy of BUS for diagnosing IBD compared to colonoscopy with a biopsy, Fisher's exact test was performed to accommodate groups of less than five and to determine the independence of groups at a significance level (*α*) of 0.05. Finally, the accuracy of the use of the BUS imaging protocol for the diagnosis of IBD was gleaned from the analyses.

## 3 Population

Adult patients who visited gastroenterology clinics and who had been referred for abdominal ultrasound scans were invited to participate in the study. Patients were excluded if they were pregnant, younger than 18 years, or clinically unstable (for instance, patients with severely tender abdomens or unstable heart conditions). Consenting participants who were older than 18 years, diagnosed with IBD or presenting with clinical symptoms suggesting IBD, and who were scheduled for colonoscopy and biopsy were included in the study. To reduce patient travel time and accommodation expenses, the ultrasound scan was performed on the same day before the colonoscopy and biopsy.

### 3.1 Sample Size

In this 9‐month study, the data of 77 participants were captured by the sonographer. To ascertain if valid conclusions could be drawn from this sample size, Slovan's formula (Bobbitt 2023) was applied using an estimate of 96 patients who had undergone colonoscopy with biopsy in an earlier 9‐month period. At a 5% margin of error, the result of the calculation indicated that 77 participants made up an appropriate sample size for this prospective study.

## 4 BUS Imaging Protocol for IBD

To design the BUS imaging protocol, peer‐reviewed literature was reviewed to identify BUS parameters that could be used to diagnose IBD. In total, eight BUS parameters were identified in the literature and deemed appropriate for the BUS diagnosis of IBD (Table 1). Measurement criteria for each of these parameters were also established from the literature. To validate the instrument, a pilot study was performed on 10 participants. The pilot study revealed that the instrument was valid and required no further changes. Therefore, the data of the 10 pilot participants were included in the main study.

**TABLE 1:** Description of potential BUS parameters of the BUS imaging protocol for the potential diagnosis of IBD.

Potential BUS parameter	Measurement criteria	Sources
BWT	BWT of more than 3 mm	(Maconi et al. 2018; Maaser et al. 2019; Kucharzik et al. 2017)
Vascularity	Increased vascularity within a thickened bowel wall was measured using color Doppler signal (ultrasound)	(Maconi et al. 2018; Nassef et al. 2014)
Bowel wall stratification	Presence of focal or extensive disruption of bowel wall layers, which may indicate severe active bowel disease	(Maconi et al. 2018)
Haustration	Loss of haustration of the colonic bowel segment, which means loss of the small pouches of the colon, caused by sacculation	(De Voogd et al. 2021)
Fatty wrapping	Presence of hyperechoic, almost solid tissue surrounding the inflamed bowel segment. These fatty wrappings are common in active Crohn's disease	(Jauregui‐Amezaga and Rimola 2021)
Peristalsis	Lack of compressibility and lack of peristalsis. Where the bowel wall has stiffened, it leads to a lack of peristalsis and compressibility	(Jauregui‐Amezaga and Rimola 2021)
Mesenteric lymph nodes	Presence of hypoechoic, oval‐shaped, solid structures (reactive lymph nodes)	(Furfaro et al. 2021; Tavakoli et al. 2021; Maconi et al. 2018)
Free fluid	Presence of free fluid close to the inflamed bowel segment	(Maconi et al. 2018)

## 5 Application of BUS Imaging Protocol to Collect Data

**FIGURE 2:**
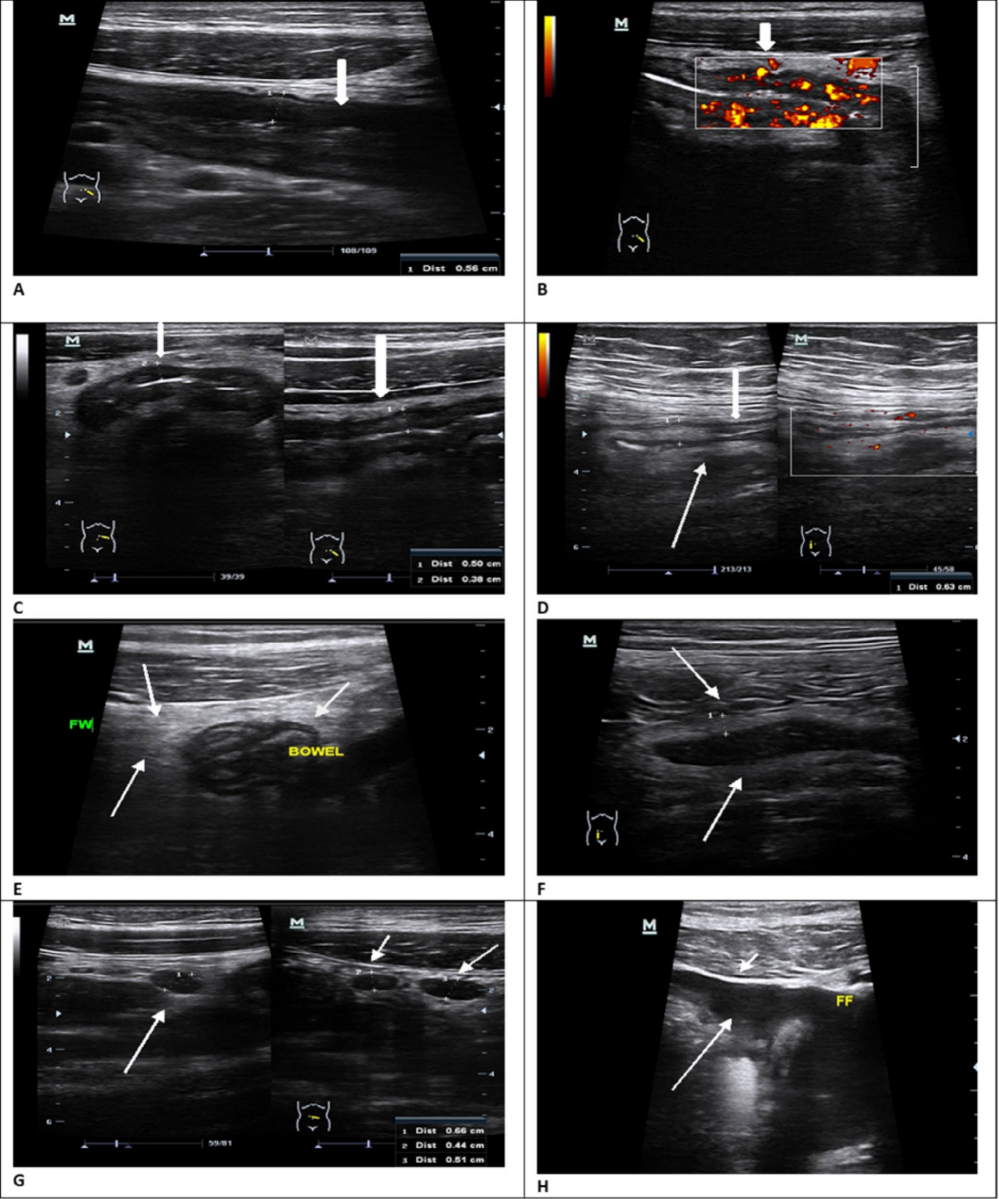
BUS images showing examples of presentations of potential BUS parameters used to diagnose suspected IBD. (A) Increased bowel wall thickness (BWT = 4.5 mm). (B) Thickened bowel wall with increased vascularity. (C) Bowel segment with loss of bowel wall stratification. (D) Thickened bowel segment with loss of haustration. (E) Fatty wrapping around a diseased bowel segment. (F) Thickened bowel loop with poor peristalsis (BWT = 6.2 mm). (G) Hypoechoic mesenteric lymph nodes (size of lymph nodes = 4.4, 6.6, and 5.1 mm in diameter). (H) Free fluid [images used with participant consent].

BUS is highly operator‐dependent. The operator needs to have a thorough knowledge and understanding of normal bowel anatomy, the fundamentals of physical principles underlying ultrasound imaging, and ultrasound techniques, which can aid in interpreting abnormal BUS findings (Atkinson et al. 2017). The sonographer in this study was a certified sonographer with more than 15 years of experience in the modality. As a qualified sonographer, she had attended an online intestinal ultrasound training program organized by the Gastroenterology Foundation of Sub‐Saharan Africa, which included a hands‐on workshop. Since attending this course, the sonographer had, over 3 years, honed her skills in performing bowel ultrasound. The radiologist who wrote the final ultrasound reports for this study has a PhD and extensive clinical experience in interpreting ultrasound images, including those of the bowel. Furthermore, to ensure consistency, data were captured using high‐resolution ultrasound and color Doppler imaging, as recommended by the EFSUMB guidelines for IBD (Saoji et al. 2024). The sonographer used the BUS imaging protocol for IBD as a guide to identify the BUS parameters in participants by assessing the sigmoid colon, descending colon, transverse colon, ascending colon, terminal ileum, proximal small bowel, and rectum. BUS was performed with a Toshiba Aplio 500 Platinum (Toshiba, Tokyo, Japan) machine and a range of transducers, including high‐frequency (5–17 MHz) linear array transducer for superficial bowel wall resolution and a low‐frequency (3–5 MHz) convex transducer to evaluate deep structures and to exclude any complications of IBD or any other gross intra‐abdominal pathology. Abdominal structures were assessed in both the longitudinal and transverse planes. The presenting parameters were recorded and saved on the PACS for interpretation by the consultant radiologist to generate a preliminary IBD diagnosis. Figure 2 presents examples of BUS images of the BUS parameters.

Several scanning perspectives were considered when performing the BUS. During the abdominal scan, the BWT was measured in the most affected intestinal segments. The BWT measurement was taken on the anterior wall and in the longitudinal plane and was measured perpendicular to the wall from the internal interface between the lumen and mucosa to the external interface between the serosa and muscularis propria (Dell'Era et al. 2023; Dolinger and Kayal 2023). Color or power Doppler ultrasound imaging was used to measure the vascularity within bowel wall segments. Increased vascularity was considered when colored pixels were consistently reproduced at the same location (Nassef et al. 2014). The mesentery was assessed to exclude or confirm lymphadenopathy and fatty wrapping. The presence of inflammatory activity was documented as positive (active) or negative (inactive). Inflammation was deemed to be present if there was bowel wall thickening (BWT > 3 mm) and if any additional established indicators or parameters of inflammation, including increased vascularity in the bowel wall, loss of bowel wall stratification, loss of haustration, fatty wrapping (mesenteric inflammatory fat), mesenteric lymph nodes, and free fluid, were present. The bowel was also evaluated for complications, including luminal stenosis, fistulas, and intra‐abdominal abscesses, which, if present, were described and recorded.

All participants underwent colonoscopies with a biopsy for histological analysis. The histological reports were reviewed to confirm IBD. To ascertain how accurate the preliminary diagnosis of IBD was for each participant, the diagnosis was compared with the confirmatory diagnosis.

## 6 Data Analysis

To compare the accuracy of the BUS parameters used for the preliminary diagnosis of IBD with the confirmatory diagnosis, SAS Version 9.2 was used to perform several statistical analyses. Descriptive statistics, including nominal frequencies and percentages, were calculated for the patients in the different diagnostic categories. Fisher's exact test was performed to determine if a statistically significant association existed between the preliminary IBD diagnosis and confirmed IBD diagnosis at a significance level of *p* = 0.05. Fisher's exact tests were chosen to accommodate categories with fewer than five participants.

The accuracy of BUS diagnosis in suspected IBD was calculated for the individual BUS parameters, as well as for the preliminary ultrasound diagnosis, using the standard accuracy equation for a diagnostic test formula (Baratloo et al. 2015; Zaborowska and Loo 2023).

## 7 Ethics Considerations

Prior to data collection, ethics approval was obtained from the Health Sciences Research Ethics Committee (UFS‐HSD2022/0610/2911), the Free State Department of Health, and the ethics research committee of the private hospital. Patient confidentiality was maintained by storing recorded data in a password‐protected file. Written informed consent was obtained from each participant before the ultrasound scan. Because ultrasound is a safe, non‐invasive, radiation‐free imaging procedure, it was not necessary to obtain permission from the Radiation Control Committee for this study.

## 8 Results

### 8.1 Biographical Information of the Sample Population

The participants were from diverse age and ethnic groups and represented both genders equally. Most participants were between 40 and 60 years old (Table 2). The sample population was represented by four ethnic groups, with the largest number of participants from the Black group (53%).

**TABLE 2:** Number of participants per age interval, gender, and ethnic groups.

Age intervals (years)			Gender		Ethnic groups			
19 to < 40	40–60	> 60 to 85	Male	Female	Black	White	Colored	Indian
9	50	18	39	38	41	26	8	2
Total	77		77		77			

### 8.2 BUS Findings

**FIGURE 3:**
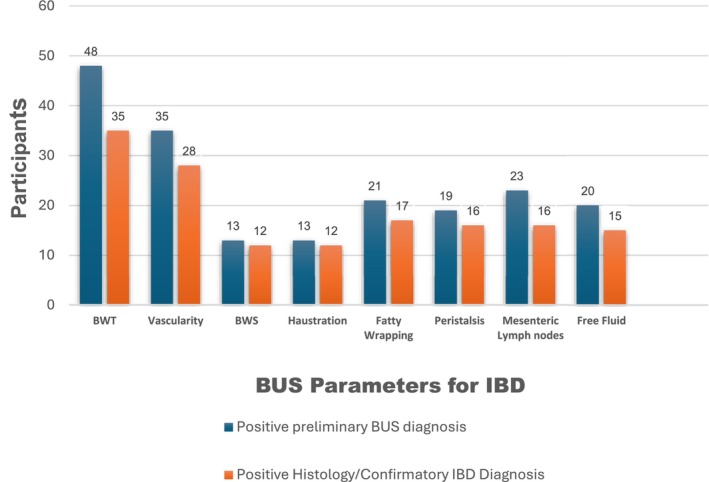
Preliminary and confirmed positive IBD diagnosis per BUS parameter category.

The preliminary positive BUS diagnosis was compared to the positive histological diagnosis of IBD to show how well the BUS imaging protocol fared in diagnosing IBD. The eight BUS parameters all indicated that more patients were positive for IBD than the histological findings revealed (Figure 3). In terms of BWT, several participants (*n* = 13) were incorrectly diagnosed with suspected IBD because they demonstrated thickened bowel walls on the scans. However, histology confirmed that these participants had other bowel pathologies, such as diverticulitis, polyps, and bowel tumors, which presented as thickened bowel walls during the BUS, which is also supported by the findings of Dell'Era et al. (2023), who found that not all patients with thickened bowel walls have IBD. This outcome indicates that IBD as well as various gastrointestinal infections such as diverticulitis, intestinal tuberculosis, and bowel tumors can exhibit similar clinical and BUS features, while these conditions typically present distinct histopathological findings (Frias‐Gomesa et al. 2021). This potential diagnostic overlap must be considered when evaluating patients with symptoms suggestive of IBD. A thorough understanding of the unique clinical, radiological, and histopathological characteristics of each condition is crucial. Additionally, employing a combination of imaging techniques and clinical assessments is essential, as BUS alone is insufficient for diagnosing gastrointestinal infections (Frias‐Gomesa et al. 2021). The primary objective of this study was to evaluate the diagnostic accuracy of BUS in detecting IBD.

The accuracy of the BUS parameters for diagnosing IBD was estimated for each of the BUS parameters. The accuracy for BWT and vascularity was above 80%; whereas the accuracy of the other BUS parameters for diagnosing IBD ranged from approximately 66% to 71% (Table 3). As a diagnostic measure for IBD, the overall accuracy of BUS using the BUS parameters was more than 80%.

**TABLE 3:** BUS parameter accuracy estimates of IBD diagnosis.

BUS parameter	Confirmed positive diagnosis	Confirmed negative diagnosis	Accuracy
BWT	35	29	83.1
Vascularity	27	35	80.5
Bowel wall stratification	12	41	68.8
Haustration	12	41	68.8
Fatty wrapping	17	38	71.4
Mesenteric lymph nodes	16	35	66.2
Peristalsis	16	39	71.4
Free fluid	15	37	67.5
Overall accuracy	35	29	81.8

Fisher's exact tests were performed to determine if a statistically significant association existed between the preliminary IBD diagnosis using ultrasound and confirmed IBD diagnosis using histological examination. The Fisher's exact tests reveal significant associations between the preliminary diagnosis of IBD and the confirmed diagnosis through histology for all the BUS parameters (two‐tailed *p* < 0.05) (Table 4). Therefore, all the null hypotheses are rejected. Fisher's exact test also shows a significant association between the preliminary diagnosis of IBD and the confirmatory diagnosis (two‐tailed *p* < 0.05).

**TABLE 4:** Contingency tests comparing patient numbers with preliminary IBD diagnoses with numbers of patients with confirmed IBD diagnoses.

BUS parameter	Preliminary IBD positive diagnosis (%)	Preliminary IBD negative diagnosis (%)	Confirmed IBD positive diagnoses (%)	Confirmed IBD negative diagnoses (%)	Fisher exact test *p*
BWT	35 (72.9)	0 (0)	13 (27.1)	29 (100)	< 0.00001
Vascularity	28 (80)	7 (20)	7 (16.7)	35 (83.3)	< 0.00001
Bowel wall stratification	12 (92.3)	23 (7.7)	1 (35.9)	41 (64.1)	< 0.0003
Haustration	12 (92.3)	23 (7.7)	1 (35.9)	41 (64.1)	< 0.0003
Fatty wrapping	17 (81)	18 (19.1)	4 (32.1)	38 (67.9)	< 0.0002
Peristalsis	16 (84.2)	19 (15.8)	3 (32.8)	39 (67.2)	< 0.00001
Mesenteric lymph nodes	16 (69.6)	19 (30.4)	7 (35.2)	35 (64.8)	< 0.0068
Free fluid	15 (75)	29 (25)	5 (35.1)	37 (64.9)	< 0.0035
Overall diagnostic comparison	35 (45.5%)	0 (0)	13 (16.9)	29 (37.7)	< 0.00001

## 9 Discussion

In this study, the use of BUS to diagnose IBD was assessed for its potential as a first‐line imaging tool to detect IBD in patients with suspected clinical symptoms. In several countries, BUS is accepted as the first line of investigation for diagnosing and monitoring IBD (Jauregui‐Amezaga and Rimola 2021; Strobel et al. 2011; Calabrese et al. 2012). The assessment entailed determining whether a combination of eight different BUS parameters gleaned from the literature could be used as a guide to diagnose IBD with BUS. The significant association between the preliminary diagnoses of IBD and the confirmatory diagnoses found by Fisher's exact tests strongly indicates that the combination of BUS parameters was relatively accurate for diagnosing IBD. For patients with a negative preliminary ultrasound diagnosis and for whom all the BUS parameters are within normal limits, a colonoscopy with a biopsy may not be necessary. In contrast, for patients for whom all the BUS parameters are outside the normal limits, the necessity of a colonoscopy and biopsy is strongly suggested.

To assess the value of using BUS parameters further to diagnose BUS, the accuracy of the parameters was calculated (Baratloo et al. 2015; Zaborowska and Loo 2023). BWT and vascularity emerged as the two parameters with accuracy values greater than 80%. This outcome is supported by Nassef et al. (2014) and Allocca et al. (2018), who report that a thickened bowel wall, in particular, is indicative of active or chronic IBD. However, according to Dell'Era et al. (2023) and Hate and Imamura (2022), not all patients who present with thickened bowel walls have IBD, which is confirmed by the 17% of patients in this study who were not diagnosed with IBD in spite of having thickened bowel walls. The thickened bowel walls of these patients could be attributed to other noninflammatory bowel conditions, such as diverticulitis, polyps, and bowel tumors. While BWT is a common feature of IBD, additional ultrasound findings such as fatty wrapping, lymphadenopathy, increased vascularity, and distinct bowel wall patterns combined with histopathological analyses can help differentiate IBD from other causes of BWT. The finding that the overall parameter accuracy exceeded 80%, with individual BUS parameter accuracies all above 66%, supports the idea that a combination of BUS parameters may enhance diagnostic accuracy when using ultrasound. This outcome aligns with the conclusions of Dell'Era et al. (2023) and Hate and Imamura (2022), who suggest that diagnosing IBD via ultrasound could be more effective when a combination of various BUS parameters is used. A significant challenge associated with IBD is differentiating between Crohn's disease and intestinal tuberculosis. Relying solely on ultrasound for diagnosis without histopathological confirmation (biopsy) risks diagnostic inaccuracies, which could lead to inappropriate treatment and exacerbate clinical outcomes. When biopsy is not an option, it is essential to adopt a multimodal diagnostic approach, which may include advanced imaging techniques such as MRE and CTE, along with non‐biopsy endoscopy, relevant laboratory testing (e.g., fecal calprotectin), and assessment of the clinical presentation. Therefore, a comprehensive, multimodal strategy that combines clinical evaluation, advanced imaging, and biomarker analysis is critical to achieve an accurate diagnosis and optimize patient outcomes when histopathological confirmation is unavailable.

## 10 Limitations of the Study

BUS has lower accuracy in specific bowel locations, such as the proximal jejunum and rectum (Frias‐Gomesa et al. 2021). The image quality of ultrasound can also be compromised by obesity and overlying bowel gas, making it technically challenging to evaluate these areas optimally. A study conducted by Hajiani et al. (2018) found that ultrasound is not an accurate imaging tool for assessing patients with ulcerative colitis because of the location of the disease and patients' body mass index (BMI). During this study, it was difficult to assess the rectum optimally with ultrasound because of overlying bowel gas and patients' body habitus. For one participant in the study, the histological procedure led to a diagnosis of a small abscess located in the rectum area, identified with the preliminary BUS scan. Although no information about the patients' BMI was gathered, most participants did not present obesity‐related impairment of image quality. Given the known limitations of ultrasound in obese patients, future studies should incorporate BMI data to enhance the robustness of diagnostic analyses.

At the time of the study, the hospital involved was the only public hospital in this city that performed colonoscopies. The sample size of the study was relatively small and limited to one hospital.

## 11 Conclusion

Because of the non‐invasive nature of BUS, it is well tolerated by patients; therefore, using BUS parameters as a sifting or screening tool should be considered in the workup for the diagnosis of IBD. Another advantage of BUS is that it can easily be used to evaluate the entire bowel segment, compared to colonoscopy, which is currently the gold standard. As a readily available and cost‐effective imaging tool, BUS is a practical technique, particularly in settings with limited resources. However, further studies are needed, including studies involving multiple centers and sonographers and a larger sample size of patients, to refine the use of BUS parameters in IBD workups. Such studies may expedite the diagnosis of IBD, decrease unnecessary use of invasive techniques, and reduce medical costs.

## Author Contributions

A.H. conducted the study and drafted the article. H.M. supervised the research study, reviewed, and gave feedback on the draft articles and finalized the article. All the authors approved the final version of the article.

## Conflicts of Interest

The authors declare no conflicts of interest.

## Data Availability

The data that support the findings of this study are available from the corresponding author upon reasonable request.
